# Anti-myelin-associated glycoprotein peripheral neuropathy as the only presentation of low grade lymphoma: a case report

**DOI:** 10.4076/1757-1626-2-6370

**Published:** 2009-07-27

**Authors:** Costantine Albany

**Affiliations:** Department of Medicine, St. Luke’s - Roosevelt Hospital, Columbia University College of Physicians and Surgeons1000 Tenth Ave New York, NY 10019USA

## Abstract

Peripheral nervous system involvement has been reported in systemic B or T cell lymphoma and may result from many mechanisms. We present a case of 59-year-old Caucasian man presented with a three years history of progressively worsening peripheral neuropathy with no obvious reason. An extensive workup revealed anti-myelin-associated glycoprotein neuropathy as the only presenting feature of low grade B cell Lymphoma.

## Introduction

Peripheral nervous system involvement has been reported in systemic B or T cell lymphoma and may result from many mechanisms. Intraneural localization of lymphoma could result in meningo-radiculopathy or mononeuropathies, or manifest as a sensory-motor polyneuropathy sometimes mimicking chronic inflammatory demyelinating polyneuropathy. We report here the case of a patient with a previously unknown NHL presenting with a progressive symmetric polyneuropathy found to have Anti-Myelin-Associated Glycoprotein (Anti MAG) peripheral neuropathy. The extensive work up revealed the diagnosis of low grade B-cell lymphoma.

## Case presentation

A 59-year-old Caucasian man presented with a three years history of progressively increasing pain, parasthesias and numbness of lower extremities bilaterally. The disease progressed to painful disabling weakness in lower extremities and later it involved his arms. There were no sphincter disturbances or back pain. The patient reports 25 lbs weight loss during this period. Otherwise his past medical history was unremarkable. He denied any history of smoking or alcohol intake and there was no significant family history.

On physical examination, the vital signs were a blood pressure (BP) of 110/80 mmHg, heart rate (HR) 78/min, respiratory rate (RR) 20/min and body temperature (T) 36.2ºC. The patient appeared alert and oriented. There were no lymph nodes palpable. Physical examination of the chest, abdomen, back and extremities were unremarkable. Neurological exam revealed evidence of bilateral lower extremity weakness with motor power 4/5 in distal muscular group, hypoactive reflexes in four extremities, along with hypoesthesia and decreased deep sensation.

**Figure 1.**
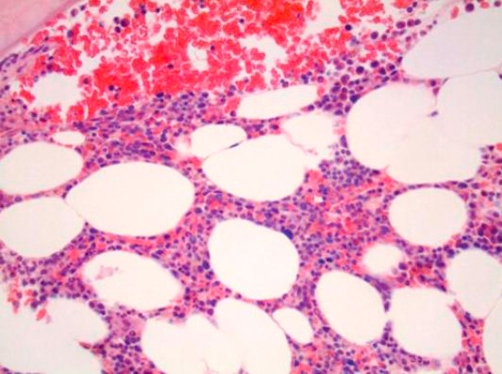
Low grade B cell Lymphoma on bone marrow biopsy (Hematoxylin and Eosin stain).

**Figure 2.**
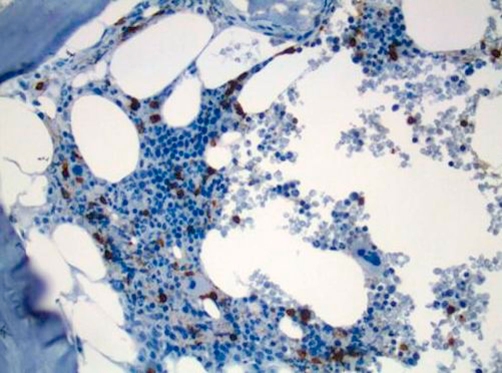
Low grade B cell Lymphoma CD20 positive on bone marrow biopsy.

A work up for peripheral neuropathy revealed normal TSH, Vitamin B12 and CPK. Monospot test, VDRL, HIV antibody, P- and C-ANCA, ANA, anti double-stranded DNA were all negative (Table 1). Electromyography (EMG) of the lower extremities showed axonal neuropathy. MRI of the spine ruled out cord lesion. CSF examinations repeatedly showed increased protein levels (80-91 mg/dl) with slightly increased white cells (<10 mm^3^) but no malignant cell identified (Table 2). Serum protein electrophoresis (SPEP) with immunofixation revealed IgM Kappa monoclonal spike. Quantative immunoglobulin analysis revealed an elevated IgM level at 313 mg/dL (the upper limit of normal is 230). Anti MAG Ab were over a million. Bone marrow biopsy and aspiration disclosed low grade CD20 positive B-cell lymphoma (Figure 1 and 2). Following the diagnosis of lymphoma patient underwent therapy with Rituximab with very good response. His neuropathy symptoms improved and his follow up Anti MAG Ab level markedly decreased (Table 3).

**Table 1.** Laboratory data

TSH	1.3 uU/ml	ANA	<1/40
CPK	34 mg/dl	ANCA	Negative
LDH	132 mg/dl	HIV	Negative
Vit B12	376 pg/ml	VDRL	Negative

**Table 2.** CSF finding

WBC	1 cells/mm^3^	Glucose	58 mg/dl
RBC	1 cells/mm^3^	Protein	94 mg/dl

**Table 3.** Anti MAG Ab follow up

T0	T6	T12
1/1,102,400	1/200,000	1/25,600

T0 upon diagnosis, T6 and T12 are after 6, 12 months follow up.

## Discussion

There are many possible causes of peripheral neuropathy. Lymphoma can affect the peripheral nervous system in 5% of patients [1]. When it does, the diagnosis can be challenging since many patients present without established diagnosis of lymphoma. In lymphoma patients most peripheral nerve complications are due to non-Hodgkin’s lymphoma (NHL). NHL can cause neuropathy by directly compressing or infiltrating nerves or by remote effects. Neuropathies could present as mononeuropathy or polyneuropathy, and may resemble an asymmetric mononeuropathy multiplex or a generalized disorder such as chronic inflammatory demyelinating polyradiculoneuropathy. Hodgkin’s lymphoma (HL), by contrast, rarely infiltrates nerves. More often, HL causes immunological disorders of the peripheral nervous system such as Guillain-Barre syndrome.

Approximately 10% of patients with peripheral neuropathy of otherwise unknown etiology have an associated monoclonal gammopathy. The hematological condition mainly associated with this entity is MGUS, but other malignancies may also occur [2,3]. Disorders such as multiple myeloma, AL amyloidosis, Waldenström’s macroglobulinemia, osteosclerotic myeloma, and lymphoma have been reported [4]. Features which suggest malignancies include weight loss, rapid progression of the neuropathy, higher levels of paraprotein (>1 g/l), elevated levels of beta-2-microglobulin and light chain proteinuria.

The anti-myelin-associated glycoprotein (MAG) neuropathy is a very rare antibody-mediated demyelinating neuropathy. The clinical picture is characterized by a distal and symmetric, mostly sensory neuropathy. Monoclonal immunoglobulin M anti-MAG antibodies are uniquely found in this condition and are believed to be pathogenic. Biopsy of nerve tissue from anti-MAG patients, demonstrates immunoglobulin M deposits at the site of MAG localization, demyelination and axonal degeneration. MAG is a Schwann cell-based glycoprotein and has been implicated as a mediator of an outside-in signaling cascade influencing the cytoskeletal integrity of axons.

Therapy in patients with anti-MAG neuropathy is directed at reducing the antibody concentration, and depleting the monoclonal B cells. Therapeutic intervention depends on the specific clinical syndrome but is generally directed at removing the autoantibodies, reducing the number of monoclonal B cells, and interfering with the effector mechanisms [5]. The recent availability of rituximab, a monoclonal antibody suppressing B-cell clones, which is not myelosuppressive and does not cause secondary malignancies, allows for early targeted intervention [6,7]. Many clinical trials, [8] have shown that in patients with IgM autoantibody associated peripheral neuropathies; rituximab treatment is followed by reduced serum concentrations of IgM, and by improvement in clinical picture [9].

In our patient, a sensorimotor demyelinating neuropathy was associated with antibodies directed against the Myelin-Associated Glycoprotein. Therefore, we present a rare case of a patient with paraproteinemic polyneuropathy who has IgM autoantibodies against Myelin-Associated Glycoprotein with underlying low grade B-cell lymphoma. The IgM type of these autoantibodies suggests that they represent all or part of the paraprotein produced by lymphoma cells. The patient responded well to rituximab treatment with significant improvement of his numbness and moderate improvement of his lower extremities weakness in three months.

## Abbreviations

ANA: Antinuclear Antibodies
ANCA: Anti-Neutrophil Cytoplasmic Antibodies
HL: Hodgkin’s lymphoma
MAG: anti-myelin-associated glycoprotein
NHL: non-Hodgkin’s lymphoma
SPEP: Serum protein electrophoresis
TSH: Thyroid Stimulating Hormone
